# Supplementary material for: Tree-ring isotopes capture interannual vegetation productivity dynamics at the biome scale
Source: Nat Commun. 2019 Feb 14;10:742. doi: 10.1038/s41467-019-08634-y (PMC6375978; doi:10.1038/s41467-019-08634-y)
Supplement: Supplementary file 1 — Supplementary Information [file 41467_2019_8634_MOESM1_ESM.pdf]

Supplementary Information

Tree-ring isotopes capture interannual vegetation productivity dynamics at the biome scale

Levesque *et al.*

Supplementary Table 1. Description of the study sites and mean climatic conditions during the growing season (April–September). VPD indicates vapor pressure deficit.

**Site**	**Species**	**Latitude** (N)	**Longitude** (W)	**Elevation** (m a.s.l.)	**Temperature** Apr.–Sept. (˚C)	**Precipitation** Apr.–Sept. (mm)	**VPD** Apr.–Sept. (kPa)
Black Rock Forest	*Liriodendron tulipifera*<br>*Quercus rubra*	41˚24'	74˚01'	300	17.6	696	0.61
Frick Creek	*Liriodendron tulipifera*<br>*Quercus rubra*	34˚40'	84˚11'	810	18.8	817	0.79
Crowley's Ridge	*Liriodendron tulipifera*	37˚04'	89˚36'	170	21.9	588	1.09
Ouachita Forest	*Quercus rubra*	34˚41'	94˚38'	650	21.9	813	1.00

Supplementary Table 2. Tree-ring chronology statistics

Site	Species	No. trees	No. cores (width)	Mean interseries correlation			Expressed population signal[1]		
				Width	$\delta^{13}C$	$\delta^{18}O$	Width	$\delta^{13}C$	$\delta^{18}O$
Black Rock Forest	*L. tulipifera*	15	30	0.46	0.84	0.55	0.93	0.96	0.86
	Q. rubra	15	30	0.43	0.80	0.61	0.92	0.95	0.89
Frick Creek	*L. tulipifera*	16	31	0.31	0.66	0.68	0.88	0.90	0.91
	Q. rubra	15	30	0.27	0.75	0.74	0.85	0.94	0.93
Crowley's Ridge	*L. tulipifera*	16	31	0.47	0.74	0.57	0.93	0.94	0.87
Ouachita Forest	*Q. rubra*	20	40	0.57	0.61	0.70	0.96	0.88	0.92

[1] The expressed population signal provides an estimate of how closely a given mean tree-ring chronology based on a finite number of trees expresses its hypothetically perfect chronology based on an infinite number of trees. Chronologies with EPS ≥0.85 are considered adequate to reflect a common signal [1].

Supplementary Table 3. Description of the sampled trees used for isotopic analysis

Site	Species	No. trees	DBH range (cm)	Height range (m)	Age range (years)
Black Rock Forest	*L. tulipifera*	5	55–67	33–36	113–136
	Q. rubra	5	31–55	15–18	132–159
Frick Creek	*L. tulipifera*	5	59–65	37–42	235–412
	Q. rubra	5	41–60	30–35	107–135
Crowley's Ridge	*L. tulipifera*	5	51–66	27–33	58–64
Ouachita Forest	*Q. rubra*	5	25–44	15–20	99–115

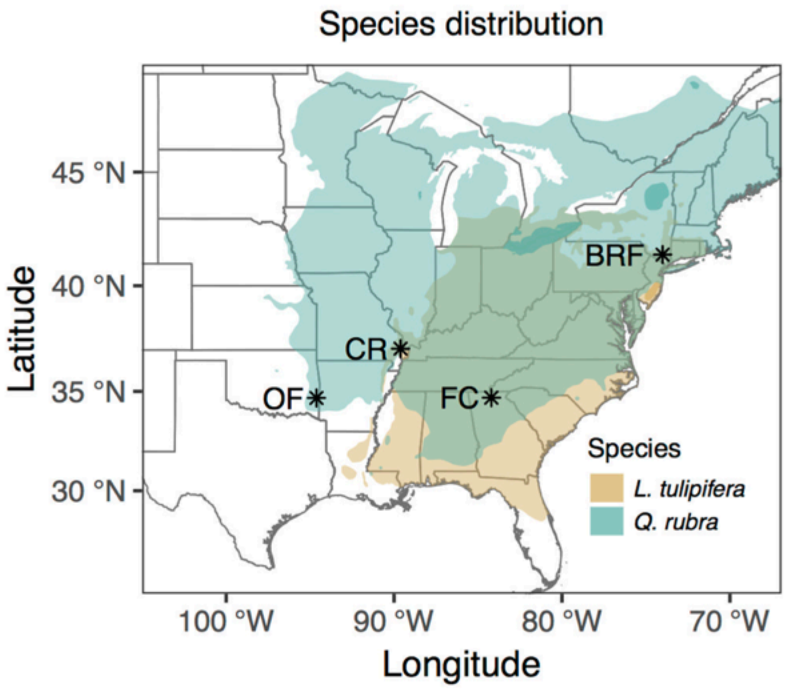

Supplementary Figure 1. Location of the study sites and distribution of *Liriodendron tulipifera* L. and *Quercus rubra* L. in eastern North America. Site abbreviations: BRF, Black Rock Forest; FC, Frick Creek; CR, Crowley's Ridge; OF, Ouachita Forest. Species distribution data are from the U.S. Geological Survey and are available online (https://www.fs.fed.us/nrs/atlas/littlefia/).

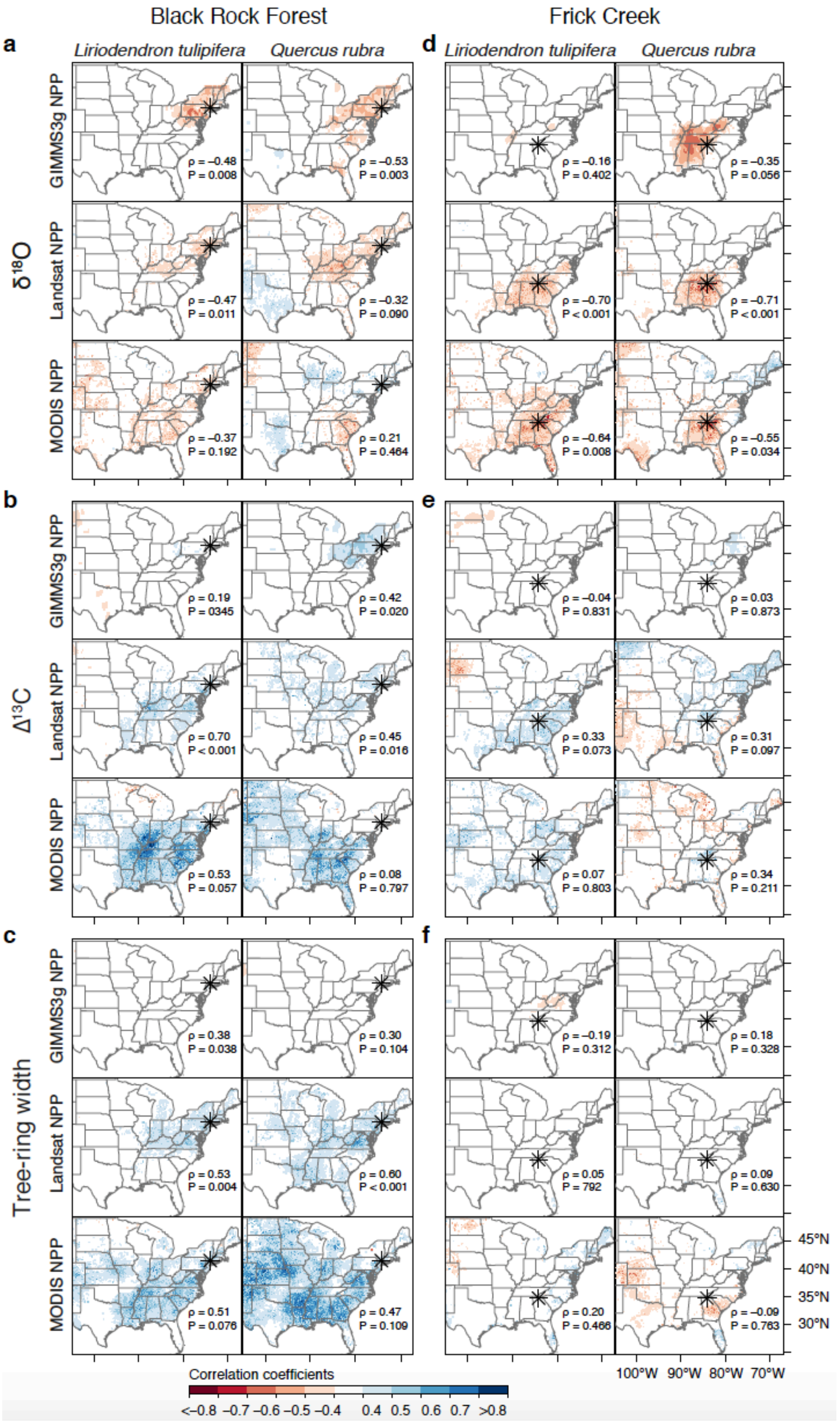

Supplementary Figure 2. Spatial field correlations between tree-ring variables of *Liriodendron tulipifera* and *Quercus rubra* measured at Black Rock Forest (**a, b, c**) and Frick Creek (**d, e, f**) and satellite NPP products. Correlations coefficients were calculated between annually resolved $\delta^{18}O$ (**a, d**), $\Delta^{13}C$ (**b, e**), and width (**c, f**) tree-ring chronologies measured at the study sites and GIMMS3g NPP (1982–2011), Landsat NPP (1986–2015) and MODIS NPP (2001–2015) datasets. Study sites are represented by stars. Site-level correlations and their significance are indicated by Spearman's rank correlation coefficient (ρ) and p-value (P).

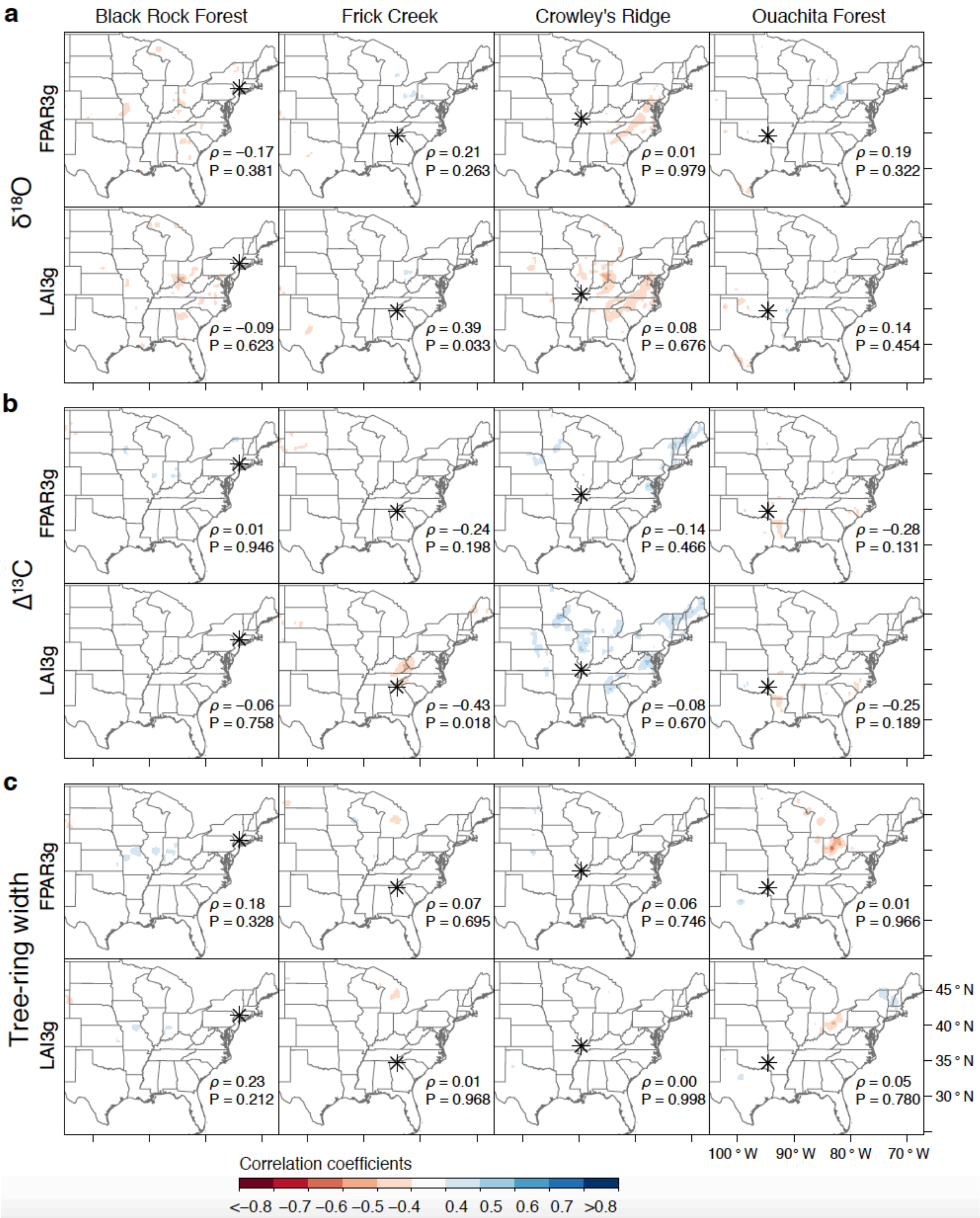

Supplementary Figure 3. Spatial field correlations between tree-ring variables and remote sensing data. Correlations coefficients for the period 1982–2011were calculated between annually resolved $\delta^{18}O$ (**a**), $\Delta^{13}C$ (**b**), and width (**c**) tree-ring chronologies measured at four sites and fraction of absorbed photosynthetically active radiation (FPAR3g) and leaf area index (LAI3g). Study sites are represented by stars. Site-level correlations and their significance are indicated by Spearman's rank correlation coefficient (ρ) and p-value (P).

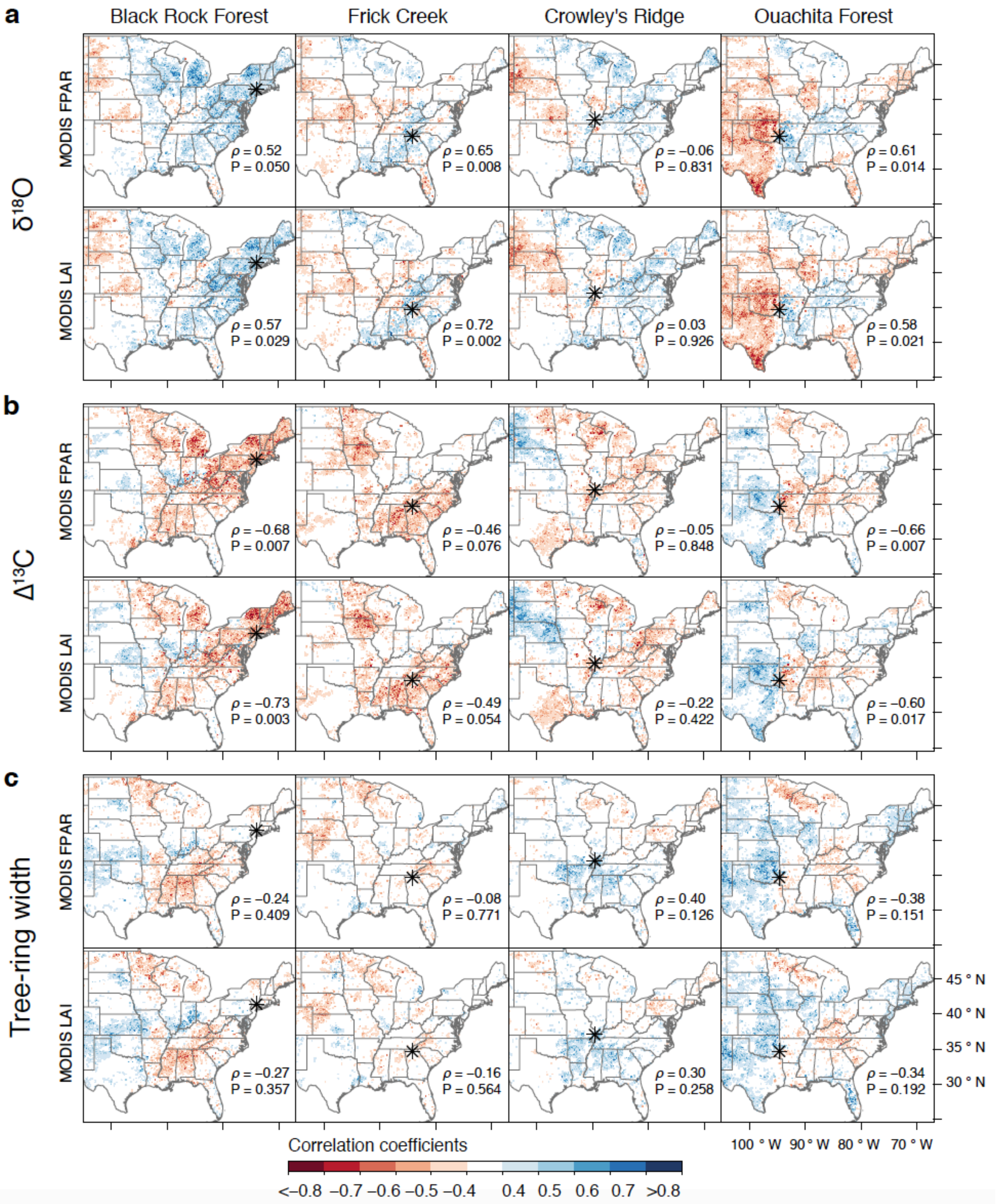

Supplementary Figure 4. Spatial field correlations between tree-ring variables and MODIS fraction of absorbed active radiation (FPAR) and leaf area index (LAI) for the period 2000–2015. Correlations coefficients were calculated between annually resolved $\delta^{18}O$ (**a**), $\Delta^{13}C$ (**b**), and width (**c**) tree-ring chronologies measured at four sites and remotely sensed MODIS FPAR and LAI data. Study sites are represented by stars. Site-level correlations and their significance are indicated by Spearman's rank correlation coefficient (ρ) and p-value (P).

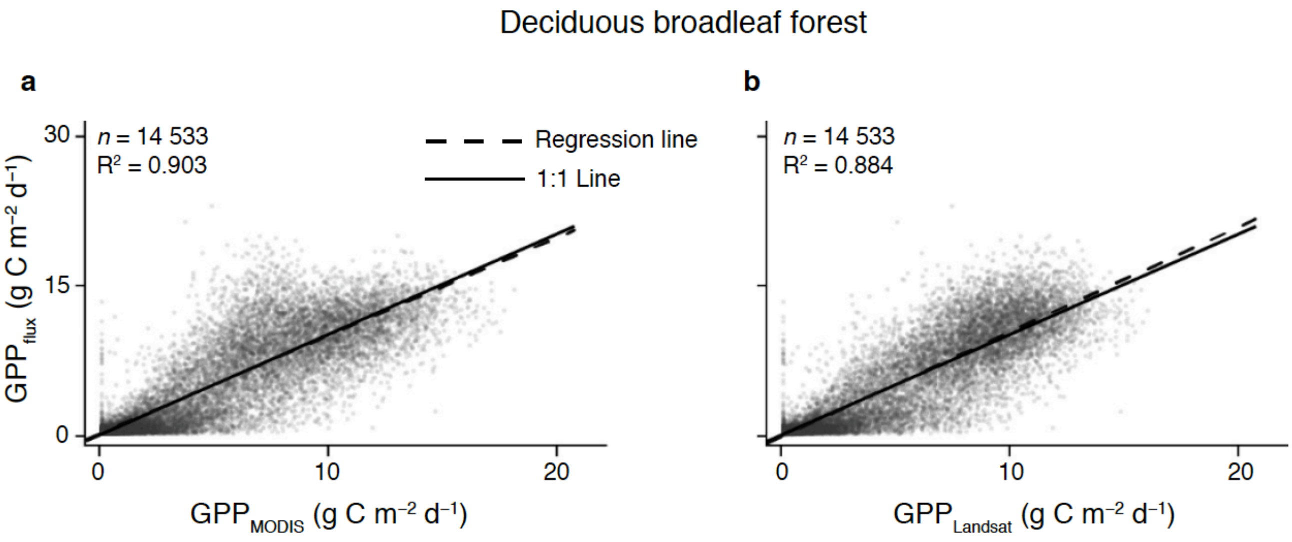

Supplementary Figure 5. MODIS gross primary production (GPP) 250m resolution (**a**) and Landsat GPP 30m resolution (**b**) relative to GPP measured at six flux towers (FLUXNET2015 dataset) over deciduous broadleaf forest in eastern United States. Each point represents an 8-day mean GPP estimate from the eddy covariance flux towers versus modeled 8-day GPP value calculated using the improved MODIS MOD17 algorithm at these flux tower sites. The main improvements of the MOD17 algorithm consist to the use of a higher resolution of land cover classification and meteorological input data specific for the conterminous United States. The description of the new terrestrial primary products and their calculations are provided in Robinson et al. (2018). The MODIS and Landsat GPP satellite datasets account for 90% and 88% of the variance from the flux tower measurements, respectively, as indicated by the R^2 values. Adapted from Robinson et al. [2].

Supplementary References

1. Wigley T. M. L., Briffa K. R., Jones P. D. On the average value of correlated time series, with applications in dendroclimatology and hydrometeorology. *J. Clim. Appl. Meteor.* **23**, 201–213 (1984).

2. Robinson N. P.*, et al.* Terrestrial primary production for the conterminous United States derived from Landsat 30 m and MODIS 250 m. *Remote Sens. Ecol. Conserv.*, doi:10.1002/rse1002.1074 (2018).
