# Supplementary material for: Tree-ring isotopes capture interannual vegetation productivity dynamics at the biome scale
Source: Nat Commun. 2019 Feb 14;10:742. doi: 10.1038/s41467-019-08634-y (PMC6375978; doi:10.1038/s41467-019-08634-y)
Supplement: Supplementary file 3 — Description of Additional Supplementary Files [file 41467_2019_8634_MOESM3_ESM.pdf]

Description of Additional Supplementary Files

Supplementary Data 1
Description: The yearly tree-ring data (tree-ring width, $\Delta^{13}C$, $\delta^{18}O$) measured at the four study sites and used for the analyses.
